# Predictive and Diagnostic Value of Serum Adipokines in Pregnant Women with Intrahepatic Cholestasis

**DOI:** 10.3390/ijerph19042254

**Published:** 2022-02-16

**Authors:** Nazan Yurtcu, Canan Soyer Caliskan, Huri Guvey, Samettin Celik, Safak Hatirnaz, Andrea Tinelli

**Affiliations:** 1 Department of Obstetrics and Gynecology, Sivas Cumhuriyet University Faculty of Medicine, Sivas 58140, Turkey; 2 Department of Obstetrics and Gynecology, Samsun Training and Research Hospital, Health Sciences University, Samsun 55270, Turkey; canansoyer@hotmail.com (C.S.C.); drsamettincelik97@gmail.com (S.C.); 3 Department of Obstetrics and Gynecology, Private Kütahya Parkhayat Hospital, Kütahya 43100, Turkey; huriguvey@gmail.com; 4 In Vitro Fertilization Unit, Medicana International Hospital, Samsun 55080, Turkey; safakhatirnaz@gmail.com; 5 Department of Obstetrics and Gynecology, Veris delli Ponti Hospital, 73020 Lecce, Italy; andreatinelli@gmail.com; 6 Department of Obstetrics and Gynecology, Division of Experimental Endoscopic Surgery, Imaging, Technology and Minimally Invasive Therapy, Vito Fazzi Hospital, 73100 Lecce, Italy; 7 Phystech BioMed School, Faculty of Biological & Medical Physics, Moscow Institute of Physics and Technology, State University, 141701 Moscow, Russia; 8 Xi’an Jiaotong University, Xi’an 710049, China

**Keywords:** intrahepatic cholestasis of pregnancy, adipokines, leptin, adiponectin, apelin, ghrelin, complications

## Abstract

The objective of this study was to assess the value of serum leptin, adiponectin, apelin, and ghrelin as biomarkers for the prediction and diagnosis of intra-hepatic cholestasis (ICP). This prospective study included pregnant women in the third trimester of pregnancy: 63 with ICP, 48 and 15 of whom had mild and severe disease, respectively, and 32 as controls. ICP women had increased median levels of serum leptin, adiponectin, apelin, and ghrelin compared to the controls (*p* < 0.05). These biomarkers meaningfully changed regarding the severity of ICP: While leptin was reduced, apelin and ghrelin were increased, and adiponectin was increased somewhat. To predict and diagnose ICP, the predictive values of serum leptin, adiponectin, and apelin need to be accepted as comparable, with moderate to high sensitivity and specificity; however, the predictive value of serum ghrelin was somewhat lower. More research is needed to clarify the potential properties of adipokines to gain acceptance as a predictive or diagnostic biomarker for ICP.

## 1. Introduction

Intrahepatic cholestasis of pregnancy (ICP) is a pregnancy specific disease and its cause has not been yet elucidated [1]. It is the second common liver disorder in pregnancy after viral hepatitis, which is often characterized by itching, liver dysfunction, and/or a high bile acid level in the late second or third trimester of pregnancy. The incidence associated with ethnicity and geographical features varies between 0.2% and 2%, and 9.2% and 15.6% in South American countries, such as Bolivia and Chile, respectively [2]. ICP has also been reported to be more common during winter months, correlated with poor selenium intake and vitamin D deficiency [3,4]. Symptoms and abnormalities in laboratory findings usually resolve soon after delivery, however may recur during subsequent pregnancy or hormonal contraception use [5]. In addition to maternal complications such as itching, nausea, loss of appetite, pain in the right upper quadrant, meconium-staining of amniotic fluid, respiratory distress, preterm delivery, and fetal death, ICP may also lead to serious fetal complications [6]. In ICP, transplacental gradients decrease fetal clearance of bile acids, resulting in the accumulation of bile acids in the fetus and amniotic fluid [7]. They have an increased risk of fetal intrauterine death, spontaneous and iatrogenic preterm birth, and the development of neonatal respiratory distress syndrome associated with bile acids entering the lungs [8].

Although the etiology of the ICP is genetic, environmental, and hormonal factors play a role in the development and aggravation of the disease [8,9,10]. In recent studies, the etiology of ICP includes inflammatory and immune factors, in association with the disruption of the immune balance in the placenta and maternal circulation [11,12].

Leptin is a polypeptide hormone that is primarily found in adipocytes, as well as detected in the hypothalamus, pituitary gland, stomach, intestine, placenta, and immune cells [13]. In addition to a well-known role in appetite control, leptin regulates the endocrine system, energy homeostasis, insulin secretion, and reproductive cycle [13]. Leptin also plays a role in the regulation of innate and adaptive immune responses due to its structural similarity with interleukins [14]. While tumor necrosis factor-α (TNF-α) and interleukin (IL)-1β increase the expression of leptin mRNA in adipose tissue, leptin increases the secretion of pro-inflammatory cytokines, such as TNF-α, IL-6, and IL-12 [15].

Adiponectin is an adipocytokine that is primarily produced and secreted by white adipose tissue. Although the main function of adiponectin is to regulate carbohydrate and lipid metabolism, the existence of its additive biological functions, such as immune modulatory, anti-inflammatory, and anti-fibrotic effects, are to be yet explored [16]. Adiponectin exerts its hepato-protective effects by blocking the activation of nuclear factor-κB and suppressing the secretion of pro-inflammatory cytokines, which is mainly released by Kupffer cells, including TNF-α and IL-1β, and enhancing the synthesis of anti-inflammatory cytokine IL-10 [2,4,6,17].

Apelin is an adipocyte-derived hormone and an endogenous peptide that has important roles in energy metabolism. APJ is bound to the G protein and is the endogenous ligands of Apelin and elabela APJ [18]. It has been determined that apelin is found in the heart, endothelium, vascular smooth muscle cells, brain, kidney, testis, ovary, liver, adipose tissue, lung, and breast [19]. Apelin is responsible for the regulation of an immune response, ensuring hemodynamic balance, angiogenesis, inotropy, glucose metabolism, development of atherosclerosis, as well as nutritional control and energy metabolism, since it is secreted from adipose tissue [18,20,21,22]. In addition, the apelin and APJ receptor system prevent liver regeneration in the liver and cause apoptosis, as well [23]. Moreover, apelin in human hepatic stellate cells enables the progression of fibrosis in cirrhosis. The expression of apelin is also increased in hepatic stellate cells under hypoxic and pro-inflammatory conditions [24].

Ghrelin is an acylated single-peptide hormone modified by fatty acid released from the gastric fundus mucosa [25]. It is the endogenous ligand of the growth hormone secretagogue receptor, stimulating the secretion growth hormone and being produced in the small intestine, colon, lung, pancreas, kidney, pituitary, and hypothalamus, with the exception of the stomach [26,27]. Major functions of ghrelin involve the secretion of the growth hormone, stimulation of appetite and eating, regulation of gastric acid secretion and motility, and control of pancreatic secretion; it is also responsible for the control of glucose metabolism, adiposity, blood pressure, reproduction, memory, and reward related pathways [28,29,30]. Ghrelin increases lipid storage in the liver and the risk of developing non-alcoholic fatty liver disease through various intracellular pathways [31]. However, it has a protective role against liver damage and fibrosis [32]. In addition, ghrelin influences decreasing liver inflammation by increasing adiponectin levels and decreasing free fatty acids and pro-inflammatory cytokines [33].

Evaluating the possible roles of molecules mentioned above, in the physiological and pathophysiological states of pregnancy, authors thought that measurements of serum leptin, adiponectin, apelin, and ghrelin in pregnant women with ICP may provide meaningful supports for their diagnostic roles in the management of ICP. Previous studies have shown that serum levels of total bile acid, aspartate transaminase, and alanine transaminase, and serum activities of alcohol dehydrogenase and aldehyde dehydrogenase are higher in women with ICP than in women without ICP, except those with other hepatobiliary diseases [34,35]. Although these indicators contain diagnostic values, they can also increase after some liver diseases, preeclampsia, chronic inflammatory diseases, and infections. Recent studies have revealed that immune factors as well as inflammatory factors play a role in the pathogenesis of ICP. Thus, there may be merit to investigating serum adipokines in pregnant women with ICP. The aim of this investigation was to determine the values of serum adipokines leptin, adiponectin, apelin, and ghrelin, as biomarkers for the prediction and diagnosis of ICP and its severity.

## 2. Materials and Methods

The study was performed on pregnant women in the third trimester of pregnancy, admitted to the obstetrics service of Samsun Training and Research Hospital in Samsun, Turkey. This prospective study was conducted after the approval of the Human Ethics Committee of the University of Health Sciences (approval number KAEK 2020/3/8). The participants were enrolled consecutively for a one-year study (from March 2020 to March 2021) with an allocation ratio of 2/1 for mild or severe ICP participants/healthy participants. In addition, the study protocol conforms to the ethical guidelines of the 1975 Declaration of Helsinki as reflected in a priori approval by the institution’s human research committee. Informed consents were obtained from all participants regarding the study.

All ICP women presented itching without rash. The total serum bile acid level above 10 µmol/L was considered positive for ICP diagnosis. Those with total bile acid levels between 10 and 100 µmol/L were grouped as mild ICP and those with 100 µmol/L and above were grouped as severe ICP. After being diagnosed for ICP, patients were followed up until delivery [8].

Healthy pregnant women with a normal routine pregnancy check were included in the study as the control group and the inclusion criteria were the following: singleton pregnancy within 28–32 weeks of gestation. Pregnant women with ICD and controls were excluded when there was no permission given to participate in the research, there were missing data, congenital anomalies of fetus, multiple pregnancies, HELLP syndrome (hemolysis, high liver enzymes, and low platelet count), insulin-dependent diabetes mellitus, chronic heart failure, thyroid dysfunction and kidney disease, and psychiatric disorder. Pregnant women who used drugs and consumed alcohol and cigarettes were also excluded. Then, abnormal transaminases and cholestatic enzymes were maybe to viral hepatitis, symptomatic cholelithiasis, cholecystitis, primary sclerosing cholangitis, primary biliary cholangitis, autoimmune hepatitis, Wilson’s disease, α1-antitrypsin deficiency, and cytomegalovirus, or Epstein Barr virus infection, acute fatty liver of pregnancy, drug-induced liver disease, and HIV infection were among the exclusion criteria.

The gestational age was calculated according to the first day of the last menstrual period and/or the first trimester ultrasound (US), by crown-rump length (CRL) when appropriate. US scans were performed using a Samsung Hs70A (GE Healthcare, Austria) device, equipped with 3.5 MHz abdominal and 7 MHz transvaginal transducers. In all enrolled patients, we routinely performed a physical examination, complete blood count, routine biochemistry tests, hepatitis tests, and urine examinations. After 8 h of fasting, 5 mL of blood was taken from the antecubital vein for blood chemistry analyses, and as part of demographic and obstetric data collection, age, number of pregnancy (gravida), parity, mass index (BMI), pregnancy week after ICP diagnosis, type of delivery, birth weight of newborns, Apgar score at 5th minute, presence of meconium in amniotic fluid, the condition of hospitalization in newborn intensive care unit (NICU), was also collected.

In all the pregnant women, the blood samples were analyzed for the alanine aminotransferase (ALT), aspartate aminotransferase (AST), total bile acid, bilirubin, adiponectin, leptin, ghrelin, and apelin levels. Total bile acid levels were measured spectrophotometrically using the enzymatic method in the Cobas C501 Analyzer (Roche Diagnostics, Rotkreuz, Switzerland). Other blood tests were performed within 2 h using a hematology analyzer (GEN-S; Beckman-Coulter Inc., Brea, CA, USA).

The concentrations of adiponectin in the serum were measured using commercially available enzyme-linked immunosorbent assay (ELISA) kits, in particular, the human adiponectin ELISA kit (Cloud-Clone Corp, Cat No. SEA605Hu, Wuhan, China). This kit uses a competitive inhibition enzyme immunoassay technique to detect the level of adiponectin. The adiponectin levels were expressed as ng/mL. The mean sensitivity of this assay is 0.069 ng/mL.

The concentrations of ghrelin in the serum were measured using the commercially available human ghrelin ELISA kit (Cloud-Clone Corp, Cat No. CEA991Hu, Wuhan, China). This kit uses a competitive inhibition enzyme immunoassay technique to detect the level of ghrelin. The ghrelin levels were expressed as pg/mL. The mean sensitivity of this assay is 4.87 pg/mL.

The concentrations of leptin in the serum were measured using commercially available chemiluminescence immunoassay (CLIA) kits, in particular, the human leptin CLIA kit (Elabscience Company, Cat No. E-CL-H0112, Houston, TX, USA). This kit uses a sandwich-chemiluminescence immunoassay technique to detect the level of leptin. The leptin levels were expressed as pg/mL. The mean sensitivity of this assay is 18.75 pg/mL.

The concentrations of apelin in the serum were measured using the commercially available human apelin ELISA kit (Cloud-Clone Corp., Cat No. CED066Hu, Wuhan, China). This kit uses a competitive inhibition enzyme immunoassay technique to detect the level of apelin. The apelin levels were expressed as pg/mL. The mean sensitivity of this assay is 8.25 pg/mL.

The enzymatic reactions were quantified in an automatic microplate photometer (TECAN-Sunrise, Austria GmbH, Grödig, Austria). All assays were conducted according to the manufacturer’s instructions. The samples, which have shown higher concentrations, were diluted, and measured in duplicate.

Statistical analyses were done by using IBM SPSS Statistics v23 (USA). After a normality test, continuous variables were described by the median (interquartile range (IQR): 1st quartile–3rd quartile). Categorical variables were described by numbers and percentages. The Kruskal–Wallis test with the post hoc Mann–Whitney and chi-square test were used. The receiver operating characteristic (ROC) curve analysis was used to determine the cut-off values of the parameters. *p* values less than 0.05 and 0.01 were considered as statistically significant. Significance values have been adjusted by the Bonferroni correction for multiple tests.

## 3. Results

A total of 95 pregnant women were enrolled in the study, including 63 participants with ICP and 32 participants with healthy pregnancies. The selected clinical and laboratory parameters in all groups are presented in Table 1 and Table 2.

There was no significant difference among the healthy and mild and severe ICP women age, gravida, parity, and BMI (*p* > 0.05). The median values of gestational age at birth and birth weight in the mild and severe ICP women were found to be significantly lower than the controls (*p* < 0.05). The Apgar score at 5 min in the severe ICP women was significantly lower than those of the mild ICP women and controls (*p* < 0.05). There were no significant differences between the mild ICP women and the controls with regard to the median Apgar score at 5 min (*p* > 0.05).

The rates of meconium-staining of the amnion and admission to NICU were found to be significantly higher in severe ICP women compared to mild ICP women and controls (*p* < 0.05). However, these rates were found to be similar in mild ICP women and the controls (*p* > 0.05).

Comparison of laboratory parameters in pregnant women with mild and severe ICP and the controls displayed in Table 2. In the pregnant women with ICP, 48 had mild ICP and 15 women had severe ICP. The serum median levels of total bile acid, ALT, AST, and bilirubin for the pregnant women with the severe ICP were significantly higher than those with mild ICP and controls (*p* < 0.05). The serum median levels of total bile acid, ALT, AST, and bilirubin for pregnant women with mild ICP were significantly higher than those of the controls (*p* < 0.05).

The median serum levels of leptin, adiponectin, apelin, and ghrelin are displayed in Table 2. The median serum level of leptin in women with severe ICP was significantly lower than those of women with mild ICP and the controls (*p* < 0.05). The median serum level of leptin in women with mild ICP was significantly lower than that of the controls (*p* < 0.05). There was no significant difference between pregnant women with mild and severe ICP regarding the median serum level of adiponectin (*p* > 0.05). The median serum levels of adiponectin in pregnant women with mild and severe ICP were significantly higher than that of the controls (*p* < 0.05). The median serum level of apelin in pregnant women with severe ICP was significantly higher than the women with mild ICP and the controls (*p* < 0.05). The median serum level of serum apelin in pregnant women with mild ICP was significantly higher than that of the controls (*p* < 0.05). The median serum level of ghrelin in women with severe ICP was significantly higher than the women with mild ICP and the controls (*p* < 0.05).

**Figure 1:**
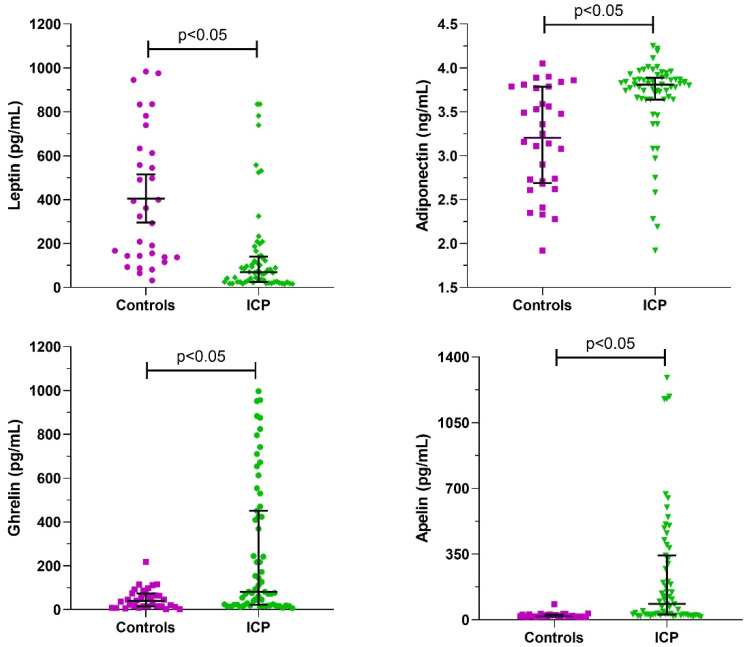
Median serum levels of leptin, adiponectin, apelin, and ghrelin levels in women with intrahepatic cholestasis (ICP) and controls. Data are expressed as median with an interquartile range and compared with the Mann–Whitney test.

A significant decrease in median level of serum leptin was detected, while there were significant increases (*p* < 0.05) in median levels of adiponectin, apelin, and ghrelin in the pregnant women with ICP compared to the controls (Figure 1).

**Figure 2:**
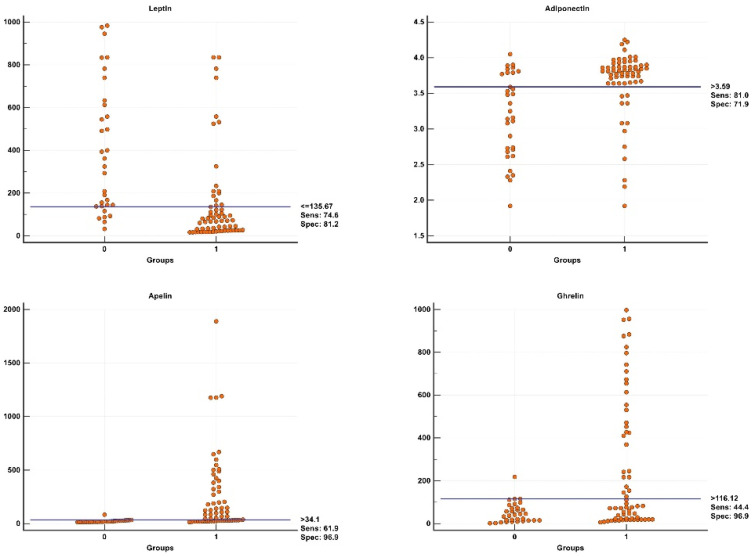

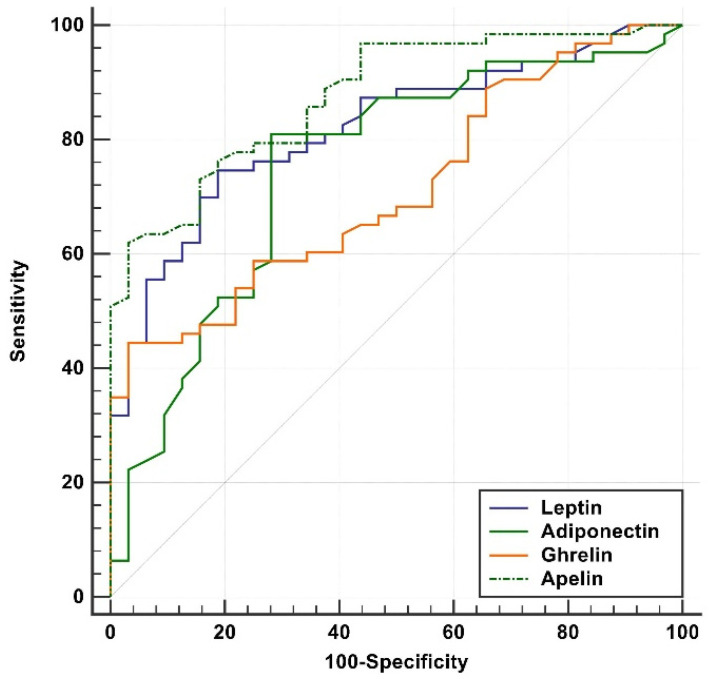
In women with or without ICP, scatter dot graphs of serum leptin (area under curve (AUC) value of 0.819 with a *p* value of *p* < 0.05), adiponectin (AUC value of 0.751 with a *p* value of *p* < 0.05), apelin (AUC value of 0.876 with a *p* value of *p* < 0.05), and ghrelin (AUC value of 0.712 with a *p* value of *p* < 0.05) with significant cut-off values (horizontal line) presented with optimal sensitivity and specificity values. Comparisons of ROC curves of serum leptin, adiponectin, apelin, and ghrelin. There is no significant difference among their area under the ROC curves (*p* > 0.05) except those of the serum apelin and ghrelin (*p* < 0.05). Overall, the predictive values of leptin, adiponectin, and apelin could be accepted as comparable.

To assess the predictive values of studied biomarkers leptin, adiponectin, apelin, and ghrelin, ROC curve analyses were performed in women with or without ICP (Figure 2). The ROC analysis of leptin data revealed a significant cut-off value ≤135.67 pg/mL with a sensitivity and specificity of 74.6% and 81.2%, respectively (*p* < 0.05). The ROC analysis of adiponectin data revealed a significant cut-off value >3.59 ng/mL with a sensitivity and specificity of 81.0% and 71.9%, respectively (*p* < 0.05). The ROC analysis of apelin data revealed a significant cut-off value >34.1 pg/mL with a sensitivity and specificity of 61.9% and 96.9%, respectively (*p* < 0.05). The ROC analysis of ghrelin data revealed a significant cut-off value >116.12 pg/mL with a sensitivity and specificity of 44.4% and 96.9%, respectively (*p* < 0.05). During the comparisons of ROC curves of serum leptin, adiponectin, apelin, and ghrelin (Figure 2), there was no significant difference among their area under curve (AUC) of the ROC analyses (*p* > 0.05) except those of the serum apelin and ghrelin (*p* < 0.05). Overall, the predictive values of leptin, adiponectin, apelin could be accepted as comparable. Figure 2 demonstrates the scatter dot graphs of serum leptin (AUC value of 0.819 with a *p* value of *p* < 0.05), adiponectin (AUC value of 0.751 with a *p* value of *p* < 0.05), apelin (AUC value of 0.876 with a *p* value of *p* < 0.05), and ghrelin (AUC value of 0.712 with a *p* value of *p* < 0.05) with significant cut-off values (horizontal line) presented with optimal sensitivity and specificity values. The scatter graphs highlight the number of cases with false positive and negative biomarker values.

## 4. Discussion

The potential roles of serum leptin, adiponectin, apelin, and ghrelin, as biomarkers for the prediction and diagnosis of ICP, were assessed with significant results: While serum leptin reduced, adiponectin, apelin, and ghrelin increased. These biomarkers revealed meaningful changes concerning the severity of ICP: While leptin was reduced, apelin and ghrelin were increased, and adiponectin was increased somewhat. For the diagnosis of ICP, the predictive values of serum leptin, adiponectin, and apelin need to be accepted as comparable, with moderate to high sensitivity and specificity, even if the predictive value of serum ghrelin was somewhat lower when compared to the other biomarkers. Based on the data in this study, the serum leptin, adiponectin, apelin, and ghrelin measurements in pregnant women with ICP could provide meaningful clues for their diagnostic roles in the prediction, diagnosis, and management of mild and severe ICP.

In a cross-sectional study, results were obtained suggesting that the leptin/adiponectin ratio increases in preeclampsia and that the imbalance between adipocytokines may play a role in the pathogenesis of preeclampsia [36]. Although adiponectin and leptin seem to be the main adipocytokines involved in the pathogenesis of GDM in the last review, their direct relationship to the pathophysiology of GDM is not clear and further studies are needed [37].

In 2013, Wikström et al. reported that there will be an increase in the incidence between ICP and both GDM and preeclampsia [38]. The relationship of ICP with GDM and preeclampsia has been evaluated and found in many studies recently. However, there are few studies on the relationship of ICP with GDM and preeclampsia, and they state that there is a lack of reliable data [37,39].

Recent studies have demonstrated that ICP is an inflammatory process specific to pregnant women with ICP, since pro-inflammatory cells and cytokines are dominant, the response of the T helper cell type-1 is increased and the process continues by means of triggering the inflammatory reaction in liver by bile acids [40,41].

Initially, adiponectin was thought to be produced only by adipose tissue. However, it was later proven from different research groups that adiponectin is expressed in other tissues such as human and murine osteoblasts, liver parenchyma cells, myocytes, epithelial cells, and placental tissue [42]. Adiponectin levels were significantly higher in the ICP group compared to healthy pregnant women, considering that Adiponectin has anti-steatosis, anti-inflammatory, and anti-fibrotic effects on liver [43,44,45]. In the study of Masaki et al. conducted on obese mice, the response to an adiponectin treatment was evaluated after causing liver damage with lipopolysaccharide [16]. As a result, they found that the adiponectin treatment decreased the elevated serum transaminase levels, hepatic necrotic and apoptotic variations, and hepatic TNF-α level after liver injury [16]. Based on this information, adipokine levels have been expected to be low in ICP in which the inflammatory mechanisms are significant. However, it has been stated that there is an increase in cholestatic diseases and the excretion of adiponectin from the body by bile is shown as the reason for this increase [46]. Another mechanism related to the elevated levels of adiponectin is that bile acids increase the release of adipokine from adipocytes in cholestatic diseases [47]. In the study conducted by Salman et al., patients with cholestasis-related cirrhosis had higher adiponectin levels than those with cirrhosis due to other factors [48]. However, differently from the previous study, an investigation including 50 pregnant women with ICP, 50 healthy pregnant women revealed that serum adiponectin levels did not demonstrate any significant change between groups [49].

In the our study, the high adiponectin levels in ICP, similarly to other cholestatic diseases, are comparable to the literature data, and this data highlights that this investigation is the first on adiponectin levels in ICP and can enrich the scientific literature on this topic. Leptin, on the other hand, is a hormone that is also released from adipocytes and exerts opposite effects to adiponectin in many ways. Leptin releases fatty acid and triglycerides from adipose tissue while adiponectin provides storage; while leptin has pro-inflammatory properties, the anti-inflammatory properties of adiponectin are prominent [50,51]. The pro-inflammatory properties of leptin are similar to those of acute phase reactants. TNF-α and IL-1 increase the leptin expression [52]. In the study findings, in contrast to this information, leptin levels were detected to be significantly lower in pregnant women with ICP, when compared to the control group and those with severe ICP, when compared to mild cases, and even if in the case where the ICP becomes severe, then the levels of Leptin decrease further.

Ben-Ari et al. evaluated patients with cirrhosis due to chronic hepatocellular disease, with primary biliary cirrhosis due to cholestasis (PBS) compared to a healthy control group [53]. Serum leptin levels were compared in both groups, and leptin levels were significantly lower in the PBS group, when compared to the other group and was found that there was no significant difference between the hepatocellular cirrhosis group and control group in terms of leptin levels. Similarly, Suarez et al. performed a study including 30 PBS patients and 29 healthy controls, the leptin levels in the PBS group were significantly lower than the control group [54]. However, it has been demonstrated that leptin levels have been shown to increase when the level of fibrosis increases. In the study of Rieger et al. including 37 PBS and 37 healthy controls, leptin levels were compared between these two groups and its association with the severity of the disease was investigated [55]. As a result, leptin levels were found to be significantly lower in the PBS group, however no association was detected between the severity of the disease and leptin levels. Although PBS is a disease that progresses with T cell infiltration, they believed that the low leptin levels were considered to be related to long lasting development of cholestasis-associated malabsorption, and consequently adipose tissue, which is a source of leptin, and may have an association with hypoplasia. Nevertheless, the ICP is not a chronic disease as PBS, and it did not cause weight loss in pregnant women in our study group.

In the study of Pizarro et al., comparing Zucker rats having obesity (due to an inactive saturation mechanism associated with deficiency in leptin production or dysfunctional leptin receptor) with normal rats, it can be understood why the leptin level was found to be low in cholestasis [56]. In this study, it was seen that bile flow decreased in Zucker rats compared to normal rats and the bile acid level increased in the serum. The decrease expression of the Mrp2 membrane protein in the ratio of 60–80% was shown as a reason, which is responsible for the transport of bile salt and organic anions and most of the bile production. Authors could explain the lower leptin levels in the ICP group with the fact that leptin deficiency decreases the expression of the Mrp2 protein.

Apelin is a cytokine that is also released from adipose tissue, such as adiponectin and leptin. Apelin has pro-inflammatory, pro-apoptotic, and fibrotic effects on the liver [23]. In the authors’ results, the levels of apelin were found to be significantly higher in pregnant women with ICP, and it was found to be higher in pregnant women with severe ICP, when compared to pregnant women with mild cases. Principe et al. showed that the mRNA level of apelin, and the expression of apelin and APJ receptors were higher in cirrhosis-induced rats with carbon tetrachloride in comparison to the control group [57]. When cirrhotic rats were treated with the receptor antagonist, hepatic fibrosis and vascular density were found to be decreased. In a study by Yasuzaki et al. on rats, the ligand of Jo2, an apoptosis-associated antigen of Fas, was injected intraperitoneal, and it was observed that the expression of apelin and APJ were both increased, and serious apoptotic changes were detected in the liver in examinations performed after 3 and 6 h [58]. Another experimental mice study by Chen et al. demonstrated that the apelin-APJ complex induced cholangiocyte proliferation and inflammation [59]. Melgar-Lesmes et al. evaluated, in human liver cell cultures, hypoxia and inflammatory factors [24]. They have been shown to activate the system of hepatic apelin, which creates angiogenic and fibrotic effects. The apelin/APJ system might contribute to cell damage in the inflammatory process in ICP.

Although ghrelin is not an adipokine, it is involved in maintaining appetite and metabolic functions in balance with adipokines. Ghrelin is the only hormone that has anti-inflammatory, hepato-protective, and antifibrotic effects on the liver, modified by fatty acid in the body and secreted from the gastric mucosa [32]. However, in the current study results, ghrelin levels were found to be significantly higher in the ICP group and it contradicts the mentioned hepato-protective effect. In addition, significantly higher ghrelin levels were found in severe ICP cases, when compared to mild cases. In a study conducted by Tacke et al. in patients with chronic liver disease, authors reported that ghrelin levels were significantly higher than that of the healthy control group [60]. They even reported that the level of ghrelin gradually increased as the clinical findings and cirrhosis worsened. Breidet et al. showed that while serum leptin levels increased in patients with primary biliary cirrhosis, there was a parallel decrease in serum ghrelin levels [61]. They attributed these changes to the insulin resistance observed in patients and the antagonistic effect between leptin and ghrelin in the hypothalamus. In this study, ghrelin levels were found to be high, while leptin was found to be low, and the antagonistic effect between leptin and ghrelin was preserved. In line with our study results, the ghrelin treatment reduced the ductal reaction and hepatic fibrosis in an experimental study on cholestatic mouse models [62]. They did not find any literature report examining the relationship of adiponectin and ghrelin with ICP, thus this study may contribute to the literature as the first study in this field.

There are some limitations of this study related to its design, including the allocation of one healthy pregnant woman per two ICP (mild or severe) women. This prevented parametric comparisons of our numeric data because of the small number of participants in the severe ICP women. To examine the impact of this condition to the post hoc power value of the studied biomarkers, we performed post hoc potency analyses on the serum adipokines leptin, adiponectin, apelin, and ghrelin in the control and ICP groups. For these parameters, potency values greater than 0.95 were found and we thought that the small number of study group participants did not compromise the interpretation of our clinical and adiponectin data.

## 5. Conclusions

The results of the current investigation suggest the role of adipokines leptin, adiponectin, and apelin and ghrelin during ICP, especially in severe cases. Considering the results of analyses for the determination of predictive values of these biomarkers for the diagnosis of ICP, leptin, adiponectin, and apelin, they provide comparable success compared to ghrelin, which has a lower predictive value. Overall, further investigations on adipokines may better explain the role of potential biomarkers for the prediction and diagnosis of ICP.

## Figures and Tables

**Table 1:** Baseline characteristics and clinical data of the women with ICP and controls.

	Controls (*n* = 32)	ICP Mild (*n* = 48)	ICP Severe (*n* = 15)	*p*
**Age (year)**	26.5 (24–32) ^a^	26 (23–30) ^a^	29 (24–34) ^a^	>0.05
**Gravida**	2 (2–3) ^a^	2 (1–3) ^a^	2 (2–3) ^a^	>0.05
**Parity**	1 (1–2) ^a^	1 (1–2) ^a^	1 (1–2) ^a^	>0.05
**BMI**	24 (23–27) ^a^	23 (23–26) ^a^	27 (23–29) ^a^	>0.05
**Gestational age at birth**	40 (39–40) ^a^	37 (36–37) ^b^	36 (33–36) ^b^	<0.05
**Birth weight (g)**	3500 (3300–3750) ^a^	3000 (2800–3200) ^b^	2900 (2700–3000) ^b^	<0.05
**Apgar score at 5 min**	9 (8–9) ^a^	9 (8–9) ^a^	8 (7–9) ^b^	<0.05
**Meconium staining of amnion, n (%)**				<0.05
**Absent**	30 (93.7%)	44 (91.7%)	6 (40%)	
**Present**	2 (6.3%) ^a^	4 (8.3%) ^a^	9 (60%) ^b^	
**Admission to NICU n (%)**				<0.05
**Absent**	30 (93.7%)	45 (93.8%)	10 (66.7%)	
**Present**	2 (6.3%) ^a^	3 (6.3%) ^a^	5 (33.3%) ^b^	

Data are presented as median with an interquartile range (25–75%), or number with a percentage as appropriate. Statistical analysis was performed with the Kruskal–Wallis ANOVA test followed by the Bonferroni-corrected Mann–Whitney post hoc test or chi-square tests. ICP, intrahepatic cholestasis of pregnancy. BMI, body mass index. NICU, neonatal intensive care unit. Each superscript letter (a, b) denotes significant difference when coded with a different letter among the study groups (*p* < 0.05).

**Table 2:** Comparison of laboratory parameters in women with ICP and controls.

	Controls (*n* = 32)	ICP Mild (*n* = 48)	ICP Severe (*n* = 15)	*p*
**Total bile acid (µmol/L)**	4 (3–4.7) ^a^	21.9 (12.8–30.5) ^b^	133 (127.4–139) ^c^	<0.05
**ALT (U/L)**	18 (12–22) ^a^	54 (48–63) ^b^	123 (78–168) ^c^	<0.05
**AST (U/L)**	17 (13–22) ^a^	51.5 (42.2–55.7) ^b^	105 (62–122) ^c^	<0.05
**Total bilirubin (mg/dL)**	0.33 (0.2–0.5) ^a^	2.1 (1.8–2.3) ^b^	3.7 (3.2–4.3) ^c^	<0.05
**Leptin (pg/mL)**	343 (140–628) ^a^	92 (42–196) ^b^	18 (17–26) ^c^	<0.05
**Adiponectin (ng/mL)**	3 (2.6–4.7) ^a^	3.8 (3.6–3.9) ^b^	3.8 (3–3.8) ^ab^	<0.05
**Apelin (pg/mL)**	19.3 (16.6–27.5) ^a^	46 (24.6–145) ^b^	547 (399–1174) ^c^	<0.05
**Ghrelin (pg/mL)**	39.2 (14.8–73.4) ^a^	66.3 (19–168) ^a^	742 (554–883.5) ^b^	<0.05

Data are presented as median with interquartile range (25–75%). Statistical analysis was performed with the Kruskal–Wallis ANOVA test followed by the Bonferroni-corrected Mann–Whitney post hoc test. ICP, intrahepatic cholestasis of pregnancy. Each superscript letter (a, b, c) denotes significant difference when coded with different letter among the study groups (*p* < 0.05).
